# Review of thermo-physical properties, wetting and heat transfer characteristics of nanofluids and their applicability in industrial quench heat treatment

**DOI:** 10.1186/1556-276X-6-334

**Published:** 2011-04-14

**Authors:** Gopalan Ramesh, Narayan Kotekar Prabhu

**Affiliations:** 1 Department of Metallurgical and Materials Engineering, National Institute of Technology Karnataka, Srinivasnagar, Mangalore, India

## Abstract

The success of quenching process during industrial heat treatment mainly depends on the heat transfer characteristics of the quenching medium. In the case of quenching, the scope for redesigning the system or operational parameters for enhancing the heat transfer is very much limited and the emphasis should be on designing quench media with enhanced heat transfer characteristics. Recent studies on nanofluids have shown that these fluids offer improved wetting and heat transfer characteristics. Further water-based nanofluids are environment friendly as compared to mineral oil quench media. These potential advantages have led to the development of nanofluid-based quench media for heat treatment practices. In this article, thermo-physical properties, wetting and boiling heat transfer characteristics of nanofluids are reviewed and discussed. The unique thermal and heat transfer characteristics of nanofluids would be extremely useful for exploiting them as quench media for industrial heat treatment.

## Introduction

**Figure 1:**
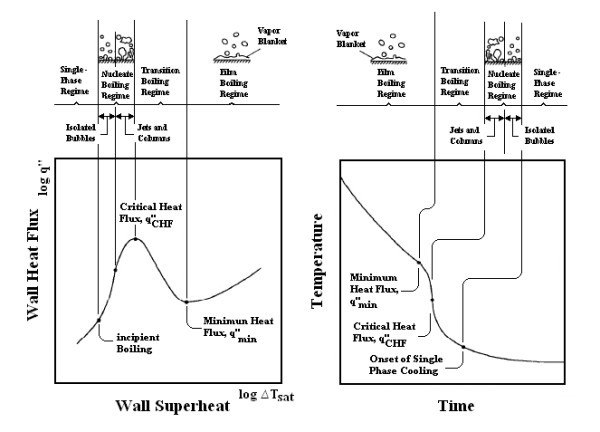
**Typical boiling (a) and temperature-time (b) curves for a hot surface quenched in a liquid bath**.

**Figure 2:**
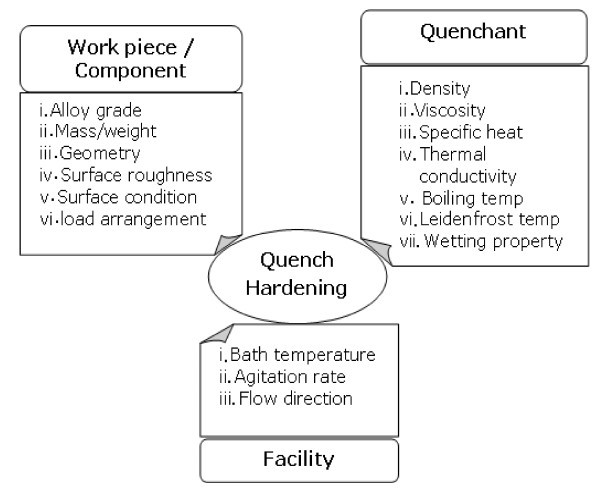
**Factors influencing the metallurgical transformation during quench hardening**.

Quench hardening is a commonly used heat treatment process in manufacturing industry to increase the service reliability of components where the material is heated to the solutionizing temperature, held for a particular period of time and then quenched into the quenching medium. Quenching during heat treatment involves simultaneous occurrence of different physical events such as heat transfer, phase transformation and stress/strain evolution, and heat transfer is the driving physical event as it triggers other processes [1]. The two phase (boiling) heat transfer is the predominant mode of heat transfer during quenching. When the hot metal submerged into the liquid pool, heat transfer is controlled by different cooling stages known as vapour blanket stage/film boiling stage, nucleate boiling stage and convective or liquid cooling stage [1-3] (Figure 1). Quenching from high temperature is enough to produce a stable vapour film around the surface of component. During this vapour blanket stage, heat transfer is very slow because the vapour film acts as an insulator and occurs by radiation through the vapour phase. Nucleate boiling starts when the surface temperature of the component drops slowly where the vapour film starts to collapse and allowing liquid to come into contact with the surface of component. The stage is characterized by violent bubble boiling as heat is rapidly removed from the part surface and maximum cooling rate is obtained. This continues till the surface temperature drops below the boiling temperature of the liquid. Quenching is a non-stationary process where the occurrence of these local boiling phenomena is a function of time and position along the surface of the component. This behaviour leads to the occurrence of a wetting front, which is the locus of the boundary between the vapour film and the occurrence of bubbles [4]. The final stage of the quenching, i.e. convection cooling occurs when the metal surface is reduced below the boiling point of quenchant. During this stage, boiling stops and heat transfer occurs directly by direct contact between the surface and liquid and the rate of heat removal is low. The important factors, which influence the heat transfer/metallurgical transformation during quench hardening, are shown in Figure 2[5]. Of all these factors listed, only a few can be changed in the heat treatment shop. The selection of optimum quenchant and quenching conditions both from the technological and economical point of view is an important consideration [5].

Water, brine solution, oil, polymer etc. are used as conventional quenching media. Water and brine solution are restricted to quenching simple shapes and steels of comparatively low hardenability because of the occurrence of intolerable distortion, warpage and quench cracks [6]. On the other hand, convective cooling in oil is less intensive due to relatively high viscosity and lower heat capacity. A variety of different quenching oils tend to show a prolonged vapour blanket stage, a short nucleate boiling stage with a much lower cooling rate, and finally a prolonged convective cooling stage with a very modest cooling rate [1]. Polymer quenchants show low cooling rate and it cannot be used with some common additives and anti oxidants. Continuous monitoring of polymer quenchant is required for optimal performance and it is not suitable for steels requiring high temperature quenching [7]. Therefore, it is necessary to develop new type of quenchants capable of producing desired property distribution, acceptable microstructure and residual stress distribution in section thicknesses of interest with avoidance of cracking and reduced distortion.

Modern nanotechnology provides new opportunities to process and produce materials with average crystallite sizes below 50 nm [8]. The unique properties of these nanoparticles are (i) size dependent physical properties, (ii) large surface area, (iii) large number density and (iv) surface structure [9]. Fluids with nanoparticles suspended in them are called nanofluids [8]. Commonly used materials for nanoparticles are oxide ceramics (Al_2_O_3_, CuO), metal carbides (SiC), nitrides (AlN, SiN), metals (Al, Cu), nonmetals (graphite, carbon nanotubes), layered (Al+Al_2_O_3_, Cu+C), PCM and functionalized nanoparticles and the base fluids includes are water, Ethylene or tri-ethylene glycols, oil, polymer solutions, bio-fluids and other common fluids [10]. There are mainly two techniques used to produce nanofluid: the single-step and two-step method. Latter method is extensively used in the synthesis of nanofluids in which nanoparticles was first produced and then dispersed in the base fluids [8]. The properly prepared nanofluids are expected to give the benefits of (i) higher heat conduction, (ii) more stability, (iii) microchannel cooling without clogging, (iv) reduced chances of erosion and (v) reduction in pumping power [11]. The addition nanoparticles to the conventional fluids result in anomalous change in thermo-physical properties of the fluid. Apart from that, the addition of nanoparticles affect the boiling behaviour at the surfaces as they fill up the discontinuity at the surfaces and probably affect the critical heat flux. Nanofluids can be considered to be the next generation heat transfer fluids as they offer exciting new possibilities to enhance heat transfer performance compared to pure liquids. They are expected to have different properties related to heat transfer as compared to conventional fluids [8]. Nanofluids offer completely different behaviour of wetting kinetics and heat removal characteristics and these characteristics could be exploited in industrial heat treatment for quenching. The present article reviews important thermo-physical properties, wetting and boiling heat transfer characteristics of the nanofluids. The importance of using nanofluids as effective quench media for hardening process during heat treatment is highlighted.

## Discussion

### Thermophysical properties of nanofluids

#### Thermal conductivity

Experiments on nanofluids have indicated that the additions of small volume fraction of nanoparticles into the base fluid have significant impact on the effective thermal conductivity of the fluid. Choi coined the term nanofluid in 1995 and proposed that the thermal conductivity of the base fluid can be increased by adding low concentration of nanoparticles of materials having higher thermal conductivity than the base fluid [12]. The transient hot wire method, the steady-state parallel-plate technique and the temperature oscillation technique are the different techniques employed to measure the thermal conductivity of nanofluids [8]. Eastman et al. showed 60% improvement in thermal conductivity by suspending 5% volume of nanocrystalline copper oxide particles in water [13]. Wang et al. observed that the effective thermal conductivity of ethylene glycol increases by about 26 and 40% when approximately 5 and 8 vol.% of Al_2_O_3 _nanopowders are added, respectively [14]. Choi measured thermal conductivity enhancement of 150% for MWCNT's dispersed in polyalphaolefin [15] and Marquis observed upto 243% increments in CNT nanofluids [16]. The summary of enhancement ratio of the thermal conductivity of water by addition of different nanoparticles is listed in Table 1[13,14,17-42]. There are no general mechanisms to explain the behaviour of nanofluids so far and the possible mechanisms for the increment of thermal conductivity of the nanofluids are as follows [43-63]:

**Table 1:** Enhancement of thermal conductivity of water on addition of nanoparticles reported in the literature [13,14,17-42].

Particle material	Particle size (nm)	Concentration (vol.%)	**Thermal conductivity ratio (*K***_**eff**_**/*K***_**f**_**)**	Remarks	Reference
Cu	100	2.50-7.50	1.24-1.78	Laurate salt Surfactant	[18]
	100-200	0.05	1.116	Spherical and square	[19]
	Not available	0.05	1.036	-	
	130-200	0.05	1.085	Spherical and square	
	75-100	0.1	1.238	Spherical and square	
	50-100	0.1	1.238	Spherical and square	
	100-300	0.1	1.110	Spherical, square, and needle	
	130-300	0.2	1.097	Spherical	
	200 × 500	0.2	1.132	Needle	
	250	0.2	1.036	Spherical, square, and needle	
Ag	60-70	0.001	1.30	30°C	[20]
			1.04	40°C	
	8-15	0.10-0.39	1.03-1.11	-	[21]
Au	10-20	0.00013	1.03	30°C (citerate reduced)	[20]
			1.05	40°C (citerate reduced)	
		0.00026	1.05	30°C (citerate reduced)	
			1.08	60°C (citerate reduced)	
Fe	10	0.2-0.55	1.14-1.18	-	[22]
Al_2_Cu	30	1.0-2.0	1.48-1.98	-	[23]
	65		1.4-1.78	-	
	104		1.35-1.60	-	
Ag_2_Al	30	1.0-2.0	1.5-2.1	-	[23]
	80		1.4-1.9	-	
	120		1.3-1.75	-	
CuO	36	5	1.6	-	[13]
	23.6	1.00-3.41	1.03-1.12	-	[24]
	23	4.50-9.70	1.18-1.36	-	[17]
	28.6	1.00-4.00	1.07-1.14	21°C	[25]
			1.22-1.26	36°C	
			1.29-1.36	51°C	
	-	1.00	1.05	-	[26]
	25	0.03-0.30	1.04-1.12	pH = 3	[27]
			1.02-1.07	pH = 6	
	29	2.00-6.00	1.35-1.36	28.9°C	[28]
			1.35-1.50	31.3°C	
			1.38-1.51	33.4°C	
	29	0-16	1.00-1.24	-	[29]
Al_2_O_3_	13	1.30-4.30	1.109-1.324	31.85°C	[30]
			1.100-1.296	46.85°C	
			1.092-1.262	66.85°C	
	38.4	1.00-4.30	1.03-1.10	-	[24]
	28	3.00-5.00	1.12-1.16	-	[17]
	60.4	1.80-5.00	1.07-1.21	-	[31]
	60.4	5.00	1.23	-	[32]
	38.4	1.00-4.00	1.02-1.09	21°C	[25]
			1.07-1.16	36°C	
			1.10-1.24	51°C	
	27-56	1.6	1.10	Sodium dodeculbenzene sulfonate	[33]
	11	1.00	1.09	21°C	[34]
			1.15	71°C	
	47		1.03	21°C	
			1.10	71°C	
	150		1.004	21°C	
			1.09	71°C	
	47	4.00	1.08	21°C	
			1.29	71°C	
	36	2.0-10.0	1.08-1.11	27.5°C	[28]
			1.15-1.22	32.5°C	
			1.18-1.29	34.7°C	
	36-47	0-18	1.00-1.31	-	[29]
SiO_2_	12	1.10-2.30	1.010-1.011	31.85°C	[30]
			1.009-1.010	46.85°C	
		1.10-2.40	1.005-1.007	66.85°C	
	-	1.00	1.03	-	[26]
	15-20	1.00-4.00	1.02-1.05	-	[21]
TiO_2_	27	3.25-4.30	1.080-1.105	31.85°C	[30]
			1.084-1.108	46.85°C	
			1.075-1.099	86.85°C	
	15	0.50-5.00	1.05-1.30	Sphere (CTAB)	[35]
	10 × 40		1.08-1.33	Rod (CTAB)	
SiC	26	4.2	1.158	Sphere	[36]
	600	4.00	1.229	Cylinder	
MWCNT	15 × 30000	0.40-1.00	1.03-1.07	-	[37]
	100 × >50000	0.60	1.38	Sodium dodecyl sulfate	[38]
	20-60 dia	0.04-0.84	1.04-1.24	Sodium dodecyl benzene 20°C	[39]
			1.05-1.31	Sodium dodecyl benzene 45°C	
	130 × >10000	0.60	1.34	CATB	[40]
	-	0-1 wt%	1.00-1.10	Gum Arabic 20°C	[41]
			1.00-1.30	Gum Arabic 25°C	
			1.00-1.80	Gum Arabic 30°C	
	-	1.00	1.07	-	[26]
	-	0.6	1.39	SDS 0.1 mass%	[42]
			1.23	SDS 0.5 m ass%	
			1.30	SDS 2 mass%	
			1.28	SDS 3 mass%	
			1.19	CTAB 0.1 mass%	
			1.34	CTAB 1 mass%	
			1.34	CTAB 3 mass%	
			1.28	CTAB 6 mass%	
			1.11	Triton 0.17 mass%	
			1.12	Triton 0.35 mass%	
			1.13	Triton 0.5 mass%	
			1.11	Triton 1 mass%	
			1.28	Nanosperse 0.7 mass%	
		0.75	1.03	CTAB 1 mass%	
			1.02	CTAB 3 mass%	
		1	1.08	CTAB 5.5 mass%	

I. *Brownian motion of nanoparticles*: The Brownian motion of nanoparticles at the molecular and nanoscale level was a key mechanism governing the thermal behaviour of nanoparticle-fluid suspensions [45]. The random motion of nanoparticles suspended in the fluid results in continuous collisions between the particles and molecules of bulk liquid thereby transport energy directly by nanoparticles. The impact of Brownian motion was more effective at higher temperatures [46]. The micro convection/mixing effect of the base fluid in the immediate vicinity of the nanoparticles caused by the Brownian motion was an important reason for the large thermal conductivity enhancement of nanofluids [47]. However, the Brownian motion contribution to the thermal conductivity of nanofluid was very small and cannot be responsible for extraordinary thermal transport properties of nanofluids [43,48-50].

II. *Liquid layering around nanoparticles*: The ordered layering of liquid molecules at the solid particle surface forms solid-like nanolayer. This layer acts as a thermal bridge between the solid nanoparticles and the base liquid and plays an important role in the enhanced thermal conductivity of nanofluids [51-54]. The effective thermal conductivity increases with increase in nanolayer thickness. Especially in small particle size range, the effects of particle size and nanolayer thickness become much more obvious, which implies that manipulating nanolayer structure might be an effective method to produce highly thermally conductive nanofluids [55]. Although the presence of an interfacial layer may play a role in heat transport, it is not likely to be solely responsible for enhancement of thermal conductivity [43]. By using molecular dynamics simulations, Xue et al. demonstrated that the layering of the liquid atoms at the liquid-solid interface does not have any significant effect on thermal transport properties [58].

III. *Nature of the heat transport in the nanoparticles*: When the nanoparticle size becomes very small, the mean free path of phonon is comparable to the size of the particle. In that case diffusive thermal transport in nanoparticles is not valid and ballistic transport is more realistic. Keblinski et al. indicated that inside the solid particles, heat moves in a ballistic manner that involves multiple scattering from the solid/liquid interface, which plays a key role in translating fast thermal transport in particles into high overall conductivity of the nanofluids. They also suggested that particles may be much closer due to Brownian motion and thus enhance coherent phonon heat flow among the particles [43]. The estimated mean free path and the transition speed of phonons in nanofluids through density functional theory indicated that the speed of phonon transport will not be affected due to the existence of nanoparticles in the low volume fraction limit [59].

IV. *Clustering of nanoparticles*: Since nanoparticles in the fluid are in Brownian motion and the Van der Waals force against gravity results in clustering of nanoparticles into percolating patterns with lower thermal resistance paths. With decreasing packing fraction, the effective volume of the cluster increases thus enhancing the thermal conductivity. Clustering may also exert a negative effect on the heat transfer enhancement particularly at low volume fraction, by settling small particles out of the liquid and creating large regions of particle free liquid with high thermal resistance [43]. Using non-equilibrium molecular dynamics simulations, Eapen et al. showed that the thermal conductivity of a well-dispersed nanofluid was enhanced beyond the 3φ Maxwell limit through a percolating amorphous-like fluid structure at the cluster interface [60]. Studies on clustering of nanoparticles in the fluids suggest varying values of thermal conductivities, i.e. enhanced, reduce and unchanged thermal conductivity of nanofluids [61-63]. Ozerinc et al. mentioned that there should be an optimum level of clustering for maximum thermal conductivity enhancement [44].

The experimentally measured thermal conductivities of nanofluids deviate from conventional models such as Maxwell, Hamilton-Crosser, Jeffery, Davis, Bruggeman, Lu and Lin model. The important factors, which control the thermal conductivity of nanofluids, are particle volume concentration, particle material, particle size, particle shape, base fluid material, temperature, additive and acidity [17,44]. Due to these complex variables and different mechanisms, the exact model for effective thermal conductivity of nanofluid is difficult. Yu and Choi have modified the Maxwell equation for the effective thermal conductivity of solid/liquid suspensions to include the effect of this ordered nanolayer [51]. Wang et al. proposed fractal model for liquid with dilute suspensions of nonmetallic nanoparticles, which involves the effective medium theory. The proposed model describes the nanoparticle clusters and their size distribution [64]. Xue presented a novel model considering the interface effect between the solid particles and the base fluid in nanofluids based on Maxwell theory and average polarization theory [65]. Jang and Choi devised a theoretical model that accounts for the role of Brownian motion of nanoparticles in nanofluid. This model also includes the concentration, temperature and size dependent conductivity [45]. By considering the particle dynamics (Brownian motion), Koo and Kleinstreuer expressed a model which consists of particle volume fraction, particle size, particle material and temperature dependence as well as properties of base liquid [46]. A comprehensive theoretical model has been developed by Kumar et al. which explains the enhancement in thermal conductivity of a nanofluid with respect to variation in particle size, particle volume fraction, and temperature [66]. Xue and Xu derived a model which consists of the thermal conductivity of the solid and liquid, their relative volume fraction, the particle size and interfacial properties [67]. Patel et al. introduced a concept of micro-convection into Kumar et al. model for predicting the thermal conductivity accurately over a wide range of particle sizes (10 to 100 nm), particle concentrations (1 to 8%), particle materials (metal particles as well as metal oxides), different base fluids (water, ethylene glycol) and temperature (20 to 50°C) [68]. By considering the effect of the interfacial layer at the solid particle/liquid interface, Leong et al. proposed a model which accounts for the effects of particle size, interfacial layer thickness, volume fraction and thermal conductivity [54]. For carbon nanotube (CNT) nanofluids, Patel et al. presented a simple model which shows linear variation of the thermal conductivity of CNT nanofluid with volume concentration [69]. Feng et al. expressed a model as a function of the thermal conductivities of the base fluid and the nanoparticles, the volume fraction, fractal dimension for particles, the size of nanoparticles, and the temperature, as well as random number. Monte Carlo technique combined with fractal geometry theory is applied to predict the thermal conductivity of nanofluids [70]. Shukla and Dhir developed a microscopic model based on the theory of Brownian motion of nanoparticles in a fluid which account size of the particle and temperature [71]. Moghadassi et al. presented a novel model based on dimensionless groups which included the thermal conductivity of the solid and liquid, their volume fractions, particle size and interfacial shell properties. The proposed model creates a non-linear relation between the effective thermal conductivity and nanoparticle volume fraction [72]. Wang et al. proposed a Novel Statistical Clustering Model to determine the macroscopic characteristics of clusters, and then, the thermal conductivity of a nanofluid [73]. Sitprasert et al. modified the Leong model inorder to predict both the temperature and the volume fraction dependence of the thermal conductivity of nanofluids for both non-flowing and flowing fluids [57]. Murugesan and Sivan developed lower and upper limits for thermal conductivity of nanofluids. The upper limit is estimated by coupling heat transfer mechanisms like particle shape, Brownian motion and nanolayer while the lower limit is based on Maxwell's equation [74]. Teng et al. proposed an empirical equation incorporating the nanoparticle size, temperature and lower weight fraction of Al_2_O_3_/water nanofluid [75]. By considering nanoparticles as liquid-like particles, Meibodi et al. expressed a model for estimation of upper and lower limits of nanofluid thermal conductivity [76].

#### Viscosity

Viscosity is an intrinsic property of a fluid that influences flow and heat transfer phenomena. The addition of nanoparticles to the base fluid shows Newtonian and/or Non-Newtonian behaviour depending on the volume percentage of particles, temperature and methods used to disperse and stabilize the nanoparticle suspension [41,77-79]. The effective viscosity of nanofluid increases by increasing concentration of particles and decreases with increase in temperature [14,41,78,80-82]. The effective viscosity of fluid containing a dilute suspension of small particles is given by Einstein's equation. Mooney extended Einstein equation to apply to a suspension of finite concentration [83]. Later Brinkman modified the Einstein equation to more generalized form [84]. However, the experimentally measured nanofluids viscosities deviate from the classical model because these models relate viscosity as a function of volume concentration only and there is no consideration of temperature dependence and particle aggregation [77]. Pak and Cho measured viscosities of the dispersed fluids with γ-Al_2_O_3 _and TiO_2 _particles at a 10% volume concentration and were approximately 200 and 3 times greater than that of water [81]. Wang et al. observed 20 to 30% increase in viscosity of water when 3 vol.% Al_2_O_3 _nanoparticles is added to water [14]. Das et al. measured the viscosity of water-based Al_2_O_3 _nanofluids at 1 and 4 vol.%. They found that the increase of viscosity with particles concentration but the fluid remains Newtonian in nature [78]. Experimental studies on CNT nanofluid by Ding et al. [41] found the shear thinning behaviour at low shear rates but slight shear thickening at shear rates greater than 200s^-1^. Kulkarni et al. investigated the rheological behaviour of copper oxide (CuO) nanoparticles of 29 nm average diameter dispersed in deionized (DI) water over a range of volumetric solids concentrations of 5 to 15% and temperatures varying from 278 to 323 K. These experiments showed that nanofluids exhibited time-independent pseudoplastic and shear-thinning behaviour. The suspension viscosities of nanofluids decrease exponentially with respect to the shear rate [79]. Similarly Namburu et al. showed the non-Newtonian behaviour at sub-zero temperatures below -10°C and Newtonian behaviour above -10°C in SiO_2 _nanofluid [77]. Chen et al. categorized the rheological behaviour of nanofluids into four groups as dilute nanofluids, semi-dilute nanofluids, semi-concentrated nanofluids, concentrated nanofluids [85]. Xinfang et al. measured the viscosity of Cu-H_2_O nanofluid by using capillary viscometers and results showed that the temperature and sodium dodecylbenzenesulfonate (SDBS) concentration are the major factors affecting the viscosity of the nano-copper suspensions, while the effect of the mass fraction of Cu on the viscosity is not as obvious as that of the temperature and SDBS dispersant for the mass fraction chosen in the experiment [86]. Recently Masoumi et al. introduces a new theoretical model for the prediction of the effective viscosity of nanofluids based on Brownian motion. This model could calculate the effective viscosity as a function of the temperature, the mean particle diameter, the nanoparticle volume fraction, the nanoparticle density and the base fluid physical properties [87].

#### Specific heat

Research work on the specific heat of nanofluids is limited compared to that on thermal conductivity and viscosity. The specific heat of nanofluid depends on the specific heat of base fluid and nanoparticle, volume concentration of nanoparticles, temperature of the fluids and the literature suggests that the specific heat of nanofluid decreases with an increase in the volume concentration and increases with temperature [88-90].

According to Pak and Cho, the specific heat of nanofluids can be calculated using the following equation [81]:(1)

Under the assumptions of local thermal equilibrium between the nanoparticles and the base fluids, Xuan and Roetzel expressed specific heat equation for nanofluid as [91](2)

Nelson and Banerjee used differential scanning calorimeter for measurement of specific heat capacity of exfoliated graphite nanoparticle fibers suspended in polyalphaolefin at mass concentrations of 0.6 and 0.3%. They found an increase in the specific heat of the nanofluid with increase in the temperature. The specific heat capacity of the nanofluid was found to be enhanced by 50% compared with PAO at 0.6% concentration by weight [88]. Zhou et al. showed that specific heat capacities of nanofluids vary with the base fluids, the size and volume concentration of nanoparticles [89]. Vajjha and Das measured the specific heat of three nanofluids containing Al_2_O_3_, SiO_2 _and ZnO nanoparticles. The first two were dispersed in a base fluid of 60:40 by mass of ethylene glycol and water and the last one in deionized water. Experiments were conducted at different particle volume concentration and different temperatures. They developed a general specific heat correlation as [90]:(3)

#### Density

The density of the nanofluids can estimated from the mixture theory [81]:(4)

where ϕ is the volume fraction of the nanoparticles, ρ_p _is the density of the nanoparticles and ρ_w _is the density of the base fluid. Sundar et al. estimated the densities of nanofluids at different temperatures. The density was found to decrease with increase in temperature [92]. Similarly Harkirat measured the density of Al_2_O_3 _nanoparticles dispersed in water using specific gravity bottles at different ranges of temperature (30 to 90°C) and different concentrations of nanofluids (1 to 4%). He observed that density of nanofluids is higher than the base fluids and increase with increase in volume fraction of nanoparticles from 1 to 4%. The density of nanofluids decreases with increase in temperature upto about 80°C. Beyond this value, densities of 1 to 4% nanofluids remained nearly constant but still were more than that of water [93].

#### Surface tension

Surface tension is defined as the force acting over the surface of the liquid per unit length of the surface perpendicular to the force. Surface tension has a significant influence on the boiling process since bubble departure and interfacial equilibrium depends on it [94]. Surface tension of nanofluids prepared by without addition of any surfactant was found to differ minimally whereas addition of surfactant during preparation of nanofluids affect significantly [78,95,96]. The surfactant behaves like an interfacial shell between the nanoparticles and base fluids and modifies the surface tension of nanofluids [97]. Surface tension decreases with increases in concentration of nanoparticle and temperature [98-100].

**Figure 3:**
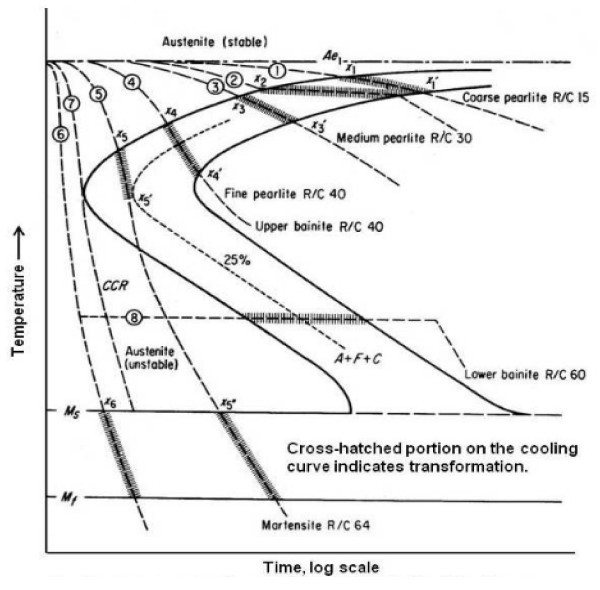
**Cooling curves superimposed on the hypothetical *I*-*T *diagram**.

**Figure 4:**
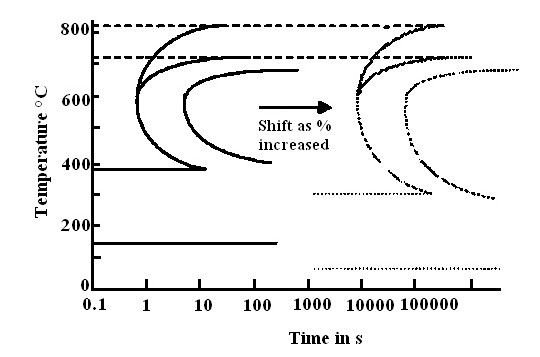
**Effect of alloying elements on TTT diagram**.

It clears from the above study, the addition nanoparticles to the base fluids would result in a change in thermophysical properties of the base fluids. A wide spectrum of microstructure and mechanical properties can be obtained for a given steel component by controlling the cooling rate (Figure 3) [101]. In order to attain the fully quenched structure (martensitic structure), the component must be quenched below the nose of the TTT curve called critical cooling rate. This critical cooling rate is not a constant for all materials and addition of alloying elements to the steel shift the nose of TTT curve (Figure 4) [102]. Therefore, the heat treaters need different types of quenching media to provide varying critical cooling rate. Table 1 shows for the same base fluid, addition different nanoparticle materials at different concentrations yield varying thermal conductivities. Jagannath and Prabhu observed peak cooling rates varying from 76°C/s to 50.8°C/s by addition of Al_2_O_3 _nanoparticles of concentration 0.01 to 4% by weight into water during quenching of copper probe [103]. The standard cooling curve analysis by Gestwa and Przyłecka observed that addition 1% of Al_2_O_3 _nanoparticles to the 10% polymer water solution results cooling speed increases from 98 to 111°C/s [104]. Babu and Kumar also observed different cooling rates with the addition of different concentration of CNT into water during quenching of stainless steel probe [105]. Further, the addition of nanoparticles not only changes the peak cooling rate but also results in change of the six cooling curve characteristics. Hence, the change in thermophysical properties of base fluids with addition of nanoparticles can be utilized to prepare fluids having different cooling properties by controlling the particle volume concentration, particle material, particle size, particle shape and base fluid. Synthesis of quenching media having varying cooling severity would greatly benefit the heat treatment industry.

### Wetting characteristics of Nanofluids

The presence of nanoparticles affects the spreading and wettability of base fluids because of additional particle-particle, particle-solid and particle-fluid interactions [106]. Two important phenomena for the enhancement of wetting behaviour of nanofluid are (i) solid like ordering of nanoparticles in the vicinity of three-phase contact region and (ii) deposition of nanoparticles during boiling. Simulations study by Boda et al. on hard spheres in a wedge-shaped cell reported formation of new layers of hard spheres between the walls of the wedge [107]. Wasan and Nikolov directly observed the particle-structuring phenomenon in the liquid film-meniscus region by using reflected-light digital video microscopy [108]. The layering arrangement of the particles gives rise to an excess pressure in the film, the structural disjoining pressure which has an oscillatory decay profile with the film thickness. A result of such a structure force is that nano-dispersions could exhibit improved spreading/wetting capabilities at a confined space [109]. The pool boiling studies on nanofluid shows deposition of porous layer of nanoparticle on the heater surface. The reason for this porous layer formation could be microlayer evaporation with subsequent settlement of the nanoparticles initially contained in it. The nanoparticles deposition improves the wettability of the surface considerably [95].

**Figure 5:**
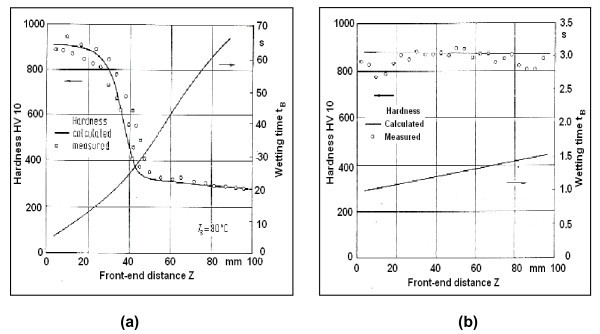
**Surface hardness profile calculated from the measured wetting time *t***_**B **_**and the specific calibration curve for the material related to the distance from the lower end of the sample and compared to the measured hardness profile**. Sample: 100Cr6 dia 25 mm × 100 mm, bath: **(a) **distilled water, **(b) **polymer solution.

During quenching, the local boiling phenomenon of quenchant leads to occurrence of a wetting front which ascends the cooling surface with a significant velocity during nucleate boiling and descends in the fluid direction during film boiling. A wetting process that occurs over a long time period of time is called non-Newtonian wetting, whereas a wetting process that occurs in a short time period or an explosion-like wetting process is termed as Newtonian wetting. A Newtonian type of wetting usually promotes uniform heat transfer and minimizes the distortion and residual stress development. In extreme cases of non-Newtonian wetting, because of large temperature differences, considerable variations in the microstructure and residual stresses are expected, resulting in distortion and the presence of soft spots [1]. Tensi has shown that the measured values indicate congruent curves for calculated hardness sample quenched in the distilled water and the total wetting time measured at the top of the sample was more than 60 s, whereas the measured hardness profile shows a continuous line in the case of sample quenched in the polymer solution having total wetting time of 1.5 s (Figure 5) [2]. Thus, the type of the wetting process significantly affects the cooling behaviour of the quenchant and hardness profile of the quenched samples. Vafaei et al. measured the contact angle of nanofluid sessile droplets and showed that the contact angle depends strongly on nanoparticle concentration and for the same mass concentration smaller size nanoparticles lead to larger changes in contact angle [110]. Sefiane et al. observed that advancing contact line velocity increases to a maximum as the concentration increases up to 1% and then decreases as the concentration is increased further. They explained that the enhanced wetting is attributed to a pressure gradient within the nanofluid which is created due to the nanoparticles forming a solid-like ordering in the fluid 'wedge' in the vicinity of the three-phase contact line and agglomeration of nanoparticles at higher concentration reduces the degree of enhanced wetting [106]. The surface wettability study by Kim et al. measured the static contact angle of sessile droplets for pure water and nanofluids on clean surfaces and nanoparticle-fouled surfaces. They found dramatic decrease of the contact angle on the fouled surfaces and concluded that the wettability was enhanced by the porous layer on the surface, not the nanoparticles in the fluid [111]. Another study by Mehta and Khandekar measured static contact angles of sessile droplets showed that the wettability of laponite nanofluid on copper substrate was indeed much better than both alumina nanofluid and pure water [112]. These studies imply that the use of nanoparticles in the conventional quenching media would result in enhancement of wettability. The enhanced wetting characteristics of nanofluids can be adopted to promote the Newtonian wetting and improve the spreading process during quench heat treatment of components.

### Boiling heat transfer characteristics of nanofluids

The alteration of thermophysical properties, especially the enhancement of the thermal conductivity, of the nanofluid and different heat transfer mechanisms are expected to have a significant effect on heat transfer characteristics. Xuan and Li [18] listed the following five reasons for improved heat transfer performance of the fluid by suspending nanophase particles in heating or cooling fluids: (i) the suspended nanoparticles increase the surface area and the heat capacity of the fluid, (ii) the suspended nanoparticles increase the effective (or apparent) thermal conductivity of the fluid, (iii) the interaction and collision among particles, fluid and the flow passage surface are intensified, (iv) the mixing fluctuation and turbulence of the fluid are intensified and (v) the dispersion of nanoparticles flattens the transverse temperature gradient of the fluid. Experiments on two phase (boiling) heat transfer of nanofluid shows different behaviour. Das et al. conducted experiments to study the pool boiling in water-Al_2_O_3 _nanofluid with different particle concentration, heater diameter and surface roughness. The results indicate that the nanoparticles have pronounced and significant influence on the boiling process deteriorating the boiling characteristics of the fluid. The deterioration in boiling performance was observed to be more drastic at a higher surface roughness. It has been observed that the shift of the curve to the right is not proportional to the particle concentration and it is strongly dependent on the tube diameter even for the similar values of surface roughness [78,113]. Zhou observed a reduction in pool boiling heat transfer of nanofluids [114]. Similarly Bang and Chang also observed that the addition of alumina nanoparticles caused a decrease of the pool nucleate boiling heat transfer. The heat transfer coefficient was decreased by increasing the particle concentration. On the other hand, CHF performance has been enhanced to 32 and 13%, respectively, for both horizontal flat surface and vertical flat surface in the pool [115]. You et al. observed the addition of nanoparticles to the water have no significant effect on nucleate pool boiling heat transfer. However, the measured pool boiling curves of nanofluids saturated at 60°C have demonstrated that the CHF increases dramatically (approx. 200% increase) compared to pure water [116]. Similarly pool boiling experiment on water-silica nanofluids by Vassallo et al. observed that no improvement in pool boiling heat transfer but the CHF increased by about three times. They observed the formation of a silica coating over the heater surface [117]. Wen and Ding observed a significant enhancement in the pool boiling heat transfer of alumina nanofluids. The enhancement increases with increasing particle concentration and reaches approximately 40% at a particle loading of 1.25% by weight [118]. Kim et al. showed 200% enhancement of CHF of nanofluids on a bare heater compared to that of pure water by increasing nanoparticle concentration. SEM images of the heater surface taken after pool boiling CHF tests revealed that CHF enhancement of nanofluids was closely related to the surface microstructure and enhanced topography resulting from the deposition of nanoparticles [119]. Kim et al. reported that the formation of the porous nanoparticle layer during the nucleate boiling is a plausible mechanism for enhancement of CHF [95]. The nucleate boiling heat transfer experiments of water-CuO nanoparticles by Liu et al. showed that the both boiling heat transfer coefficient and CHF of the nanofluids increase with the increase of the mass concentration. However, when the concentration (optimum mass concentration) is over 1 wt%, the CHF is basically close to a constant value, and the heat transfer deteriorates gradually. They also found that the boiling heat transfer of the nanofluid on the smooth surface is almost the same with that of water on the smooth surface at atmospheric pressure whereas boiling heat transfer of the nanofluids on the grooved surface increases remarkably [120]. Kathiravan et al. observed the enhancement of heat transfer coefficient during the pool boiling of water-CNT nanofluids of 0.25, 0.5 and 1.0% concentration by volume of CNT by 1.76, 1.203 and 1.20 times greater than that of heat transfer coefficient of water, respectively, at the critical heat flux. They also observed that there is no fouling over the test-section [121]. Another study by Park et al. shows that the pool boiling heat transfer coefficients of the aqueous solutions with CNTs are lower than those of pure water in the entire nucleate boiling regime but the CHF increased up to 200% as compared to that of pure water. They observed the deposition of a thin film of CNTs on the surface and decrease in the contact angle [122]. So, it is clear that the CHF during pool boiling of nanofluids increased even when the pool boiling heat transfer of nanofluid may decrease or remain unchanged.

During quench hardening process, the surface heat transfer conditions between the steel part and the quenchant are the most important factors controlling the microstructural evolution, generation of stresses and distortion [1]. Kobasko showed that very fast and uniform part cooling within the martensitic range actually reduces the probability of part cracking and distortion, while improving the surface hardness and durability of steel parts [123]. The enhanced CHF of the nanofluids during pool boiling revealed that nanofluids may be suitable for cooling at high heat flux applications [124]. According to Kim et al. the use of nanofluids can afford a significant acceleration of quenching by means of premature destabilization of film boiling due to nanoparticle deposition [125]. The quenching of 304 stainless steel probe into different concentration of nanofluids yielded varying peak heat transfer coefficient (HTC) and Grossmann severity of quenching [126]. Jagannath and Prabhu measured the interfacial peak HTC of water was 1280 W/m^2 ^K and the peak HTC decreased from 1400 to 965 W/m^2 ^K with increases in Al_2_O_3 _nanoparticle concentration from 0.01 to 4 wt% when copper is quenched [103]. Similarly Babu and Kumar observed that the peak heat flux during quenching in CNT nanofluids increases with an increase in the CNT concentration until 0.50 wt.% and starts decreasing with further increase in the CNT concentration [105]. These results suggest that for the same base fluid there is an optimum level of nanoparticle concentration to enhance/decrease the heat transfer characteristics of nanofluids. The enhancement and deterioration of pool boiling heat transfer of nanofluids could be utilized in quenching heat treatment in two ways either to promote or decrease the rate of heat transfer depending upon the section thickness of the part to be heat treated and the desired microstructure. Hence there is a need for development of nanofluids having (i) high quench severity for enhancement of heat transfer for thick sections with low quench sensitivity and (ii) low cooling severity for thin sections with high quench sensitivity [127].

### Effect of addition of nanoparticles on microstructure and mechanical properties of components

**Figure 6:**
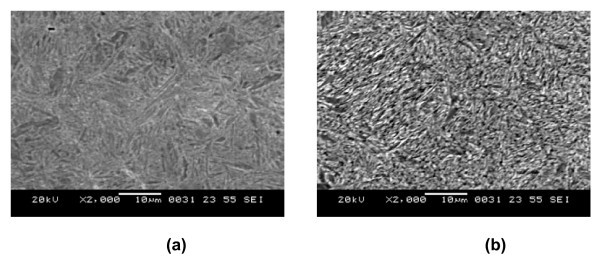
**Microstructure of AISI 1070 steel specimen (a) quenched in water (b) quenched in 0.01% nanofluid**.

**Figure 7:**
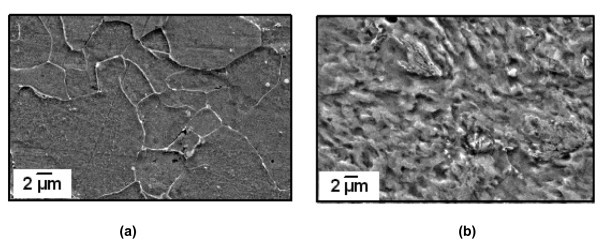
**SEM micrographs of (a) top surface of steel after cooling with water, (b) top surface of steel after cooling with nanofluid**.

The application of nanofluid in nuclear, rocket, transport and transformer industry is presently well known. It should be noted that there is no metallurgical and mechanical properties change in these applications. However, in quenching heat treatment there is a microstructural change in the component. When steel is quenched from the austentic phase, austenite may transform to ferrite, pearlite, bainite or martensite depending on the cooling rate. Phase transformations in solid state are accompanied by volume variation and transformation plasticity. Large thermal stresses and residual stresses are developed during quenching because of non-uniform cooling of parts and associated heat of metal parts released during the phase transformation. AISI 1070 specimens quenched into the water and water-Al_2_O_3 _nanofluid showed a martensitic structure (Figure 6). Finer martensitic structure was observed in 0.01% nanofluid with higher hardness [128]. Chakraborty observed the microstructure of the top surface of the steel after spray quenching with water and Water-TiO_2 _nanofluids. The cooling rate of the nanofluid was much faster than that of water resulting in ferrite-bainite structure whereas only ferrite was obtained for water quenching (Figure 7) [129]. Recent experiments with Al_2_O_3 _nanofluid by Gestwa and Przylecka observed that hardening in nanofluid results in higher impact strength in comparison to the impact strength of the samples hardened in the media without nanoparticles for both the C10 and the 16MnCr5 carburized steel samples. They also observed lowest values of the dimension changes for samples hardened and carburized in 10% polymer water solution with 1% of Al_2_O_3 _nanoscale particles [104]. It is evident that by adopting nanofluids as quenching media it is possible to obtain the desired microstructure of components and hence the required mechanical properties.

## Summary

Heat transfer and wetting kinematics are the two important phenomena during quenching that controls the final metallurgical and mechanical properties of the components. Judicious selection of quench medium is critical for obtaining optimum mechanical properties, avoiding quench cracks, minimizing distortion and improving reproducibility in hardening. The addition of nanoparticles to the conventional quenching fluid results in anomalous change in thermo-physical properties of the fluid, enhanced critical heat flux during boiling heat transfer, improved wetting characteristics and improved metallurgical and mechanical properties. By exploiting these potential advantages of nanofluids, preparation of a spectrum of quench media, known as nanoquenchants, with varying cooling severity would be extremely useful for industrial heat treatment.

## Abbreviations

CuO: copper oxide; DI: deionized; HTC: heat transfer coefficient; SDBS: sodium dodecylbenzenesulfonate.

## Competing interests

The authors declare that they have no competing interests.

## Authors' contributions

RG carried out the literature review and drafted the manuscript. KNP also carried the review and summarized the conclusions. All authors read and approved the final manuscript.
